# *Sarcina ventriculi* in an Endoscopic Ultrasound-Guided Fine Needle Aspiration of a Perigastric Lymph Node with Metastatic Pancreatic Adenocarcinoma: A Carry-Through Contaminant Bacterial Microorganism from the Stomach

**DOI:** 10.1155/2021/4933279

**Published:** 2021-12-29

**Authors:** Bharat Nandakumar, Diva R. Salomao, Nicholas A. Boire, Audrey N. Schuetz, Charles D. Sturgis

**Affiliations:** ^1^Division of Hematology, Mayo Clinic, Rochester, MN, USA; ^2^Department of Laboratory Medicine and Pathology, Mayo Clinic, Rochester, MN, USA

## Abstract

*Sarcina ventriculi* is a rare gram-positive coccus increasingly reported in patients with a history of delayed gastric emptying or gastric outlet obstruction and is sometimes seen in association with emphysematous gastritis and perforation. We report a case of a 67-year-old male who presented with epigastric pain. CT imaging and cholangiopancreatography were concerning for pancreatic neoplasia. Upper endoscopic ultrasound-guided fine needle aspiration cytology of a perigastric lymph node confirmed metastatic adenocarcinoma of pancreatic origin, and cocci arranged in a tetrad fashions characteristic of *Sarcina ventriculi* were noted. To our knowledge, this is the first reported case of *Sarcina ventriculi* in an FNA of metastatic pancreatic carcinoma in a perigastric lymph node. These organisms likely represent carry-through contaminants from the transgastric approach of the endoscopic FNA.

## 1. Introduction

First described in 1842 by John Goodsir [1], *Sarcina ventriculi* is an anaerobic, gram-positive coccus with characteristic morphological features such as basophilic staining [2], cuboidal shape [3], and tetrad arrangements [4] on hematoxylin-eosin stain. *Sarcina* organisms can survive in very low pH environments where they form their distinctive morphological features. At higher pH levels, organisms are noted to be larger and varied in shape and size [5]. *Sarcina ventriculi* has been reported to have an association with delayed gastric emptying and is sometimes implicated as a causative factor in gastric perforation [6] and emphysematous gastritis [7]. In addition, one prior publication describes the presence of this organism in patients with gastric adenocarcinoma and pancreatic adenocarcinoma [8]. We report a case of a 67-year-old male with pancreatic adenocarcinoma in whom *Sarcina ventriculi* organisms were incidentally noted on cytological examination of a perigastric lymph node biopsy performed for staging and diagnosis of adenocarcinoma of the pancreas.

## 2. Case Report

A 67-year-old man with type 2 diabetes presented approximately one year prior to diagnosis with complaints of intermittent sharp epigastric pain and bloating. In the interim, he had undergone CT imaging, upper and lower endoscopy, laboratory testing, and magnetic resonance cholangiopancreatography at outside institutions. A nonenhancing lesion of the neck of the pancreas had been noted and was favored to represent necrotizing pancreatitis. During this timeframe, he was unsuccessfully treated with a proton pump inhibitor, pancreatic enzyme replacement, gabapentin, and acetaminophen. He experienced an approximate 50-pound unexplained weight loss during this interval and ultimately sought care at Mayo Clinic.

**Figure 1:**
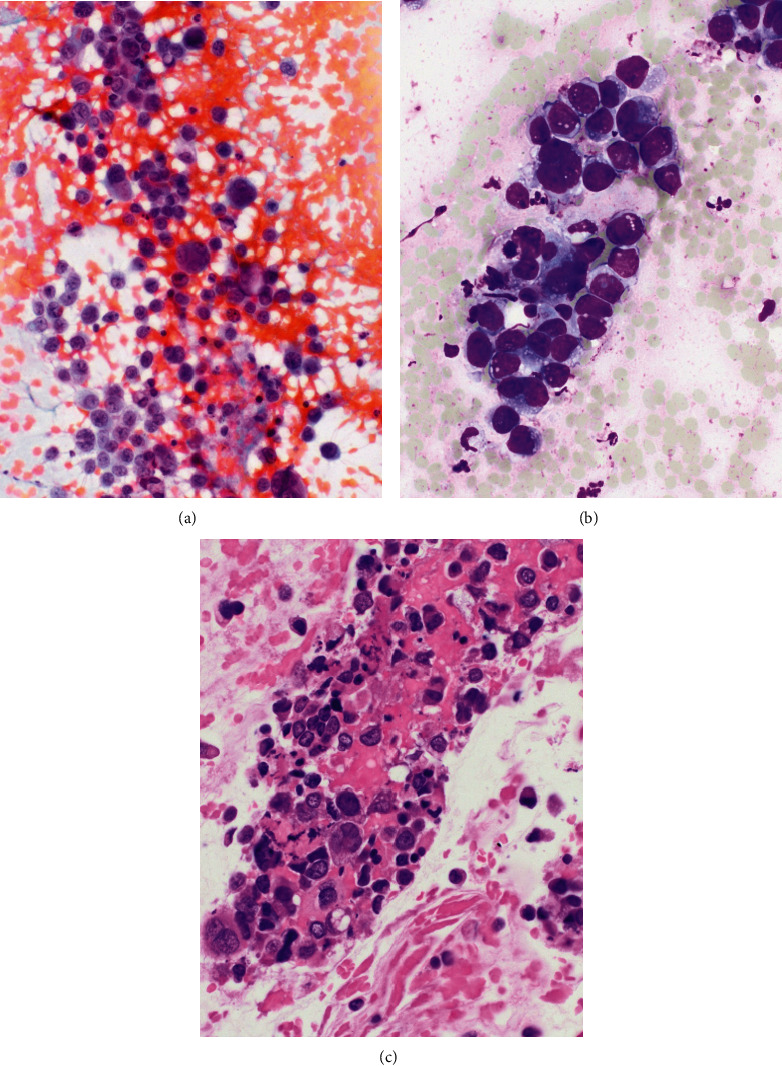
(a) High cellularity EUS FNA direct smear with loosely cohesive neoplastic cells with open chromatin and discernable nucleoli in backgrounds of blood (Papanicolaou stain, 400X). (b) Higher magnification EUS FNA direct smear with cohesive groups of high nuclear/cytoplasmic ratio malignant cells with vague acinar architecture compatible with adenocarcinoma (modified Giemsa stain, 600X). (c) Cell block preparation with background eosinophilic granular debris (necrosis) and scattered overtly cytologically malignant individual cells with hyperchromasia and anisonucleosis (hematoxylin and eosin stain, 600X).

**Figure 2:**
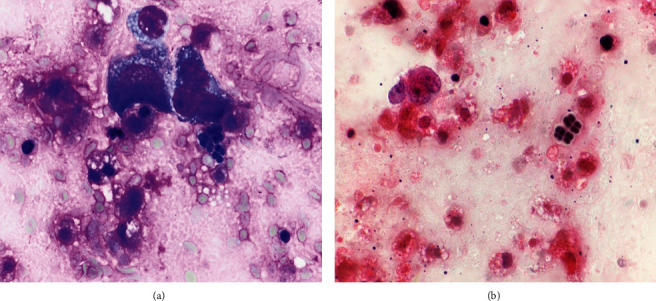
(a) As the diagnosis of malignancy was confirmed, scattered groups of microorganisms (arrows) were also identified in close proximity to some of the neoplastic cells (modified Giemsa stain, 1000X). (b) These clustered microorganisms were large and noted in tetrad arrangements with amphophilic tinctorial appearances (Papanicolaou stain, 1000X).

**Figure 3:**
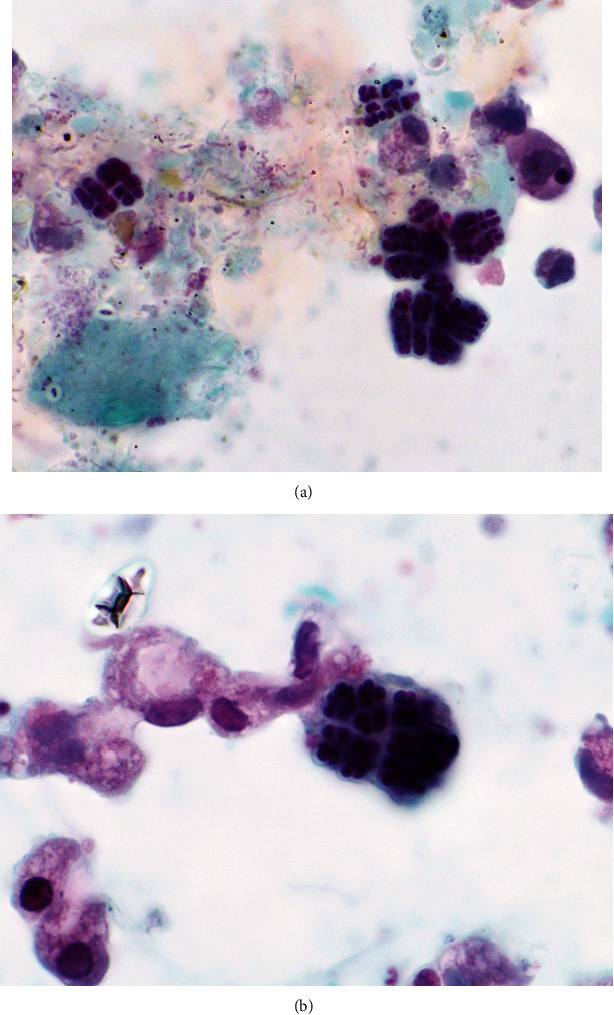
(a) In focal areas of the direct smears, numerous collections of large bacteria arranged in tetrads (*Sarcina ventriculi*) were noted. In addition, scattered smaller cocci and a few bacilli (mixed bacteria) were also identified in backgrounds with necrosis and poorly preserved nucleated cells (Papanicolaou stain, original magnification 1000X). (b) Highest power magnification depicting clustered *Sarcina* organisms with adjacent macrophages, noting comparatively clear background without accompanying acute inflammation, suggesting carry-through microorganisms from lumen of GI tract (Papanicolaou stain, original magnification 1000X).

A CT angiogram of the pancreas was conducted and demonstrated a heterogeneously enhancing pancreatic tumor involving the gastrohepatic region, the porta hepatis, the pericholecystic region, and extending along the falciform ligament. Direct invasion of the left hepatic lobe was noted, and the tumor encased the distal gastric body and antrum and partially encased the proximal duodenum. Occlusion of the splenic vein was noted near the portosystemic confluence, along with suspected invasion and large resultant collaterals. The hepatic artery and proximal splenic artery were also encased. Multiple lymph nodes and peritoneal/omental nodules were also identified. An upper endoscopic ultrasound revealed residual food in the gastric body without any signs of abnormal tissue. The first part of the duodenum was narrowed due to extrinsic compression, though patent to the endoscope, and the second portion of the duodenum and major papilla appeared normal. The findings were concordant with a partial gastric outlet obstruction. Endosonographic ultrasound-guided fine needle aspiration biopsies of an abnormal perigastric lymph node confirmed metastatic adenocarcinoma (Figure 1). In addition to malignant cells and necrosis, the fine needle aspiration cytology preparations also demonstrated admixed large coccoid bacteria with amphophilic to basophilic staining arranged in tetrad fashions, compatible with *Sarcina ventriculi* (Figures 2–3). In addition to *Sarcina* organisms, scattered small bacilli and much smaller coccoid bacteria were noted, with minimal to no accompanying inflammation identified. The mixed microbial appearance was compatible with carry-through bacteria from the lumen of the gastrointestinal tract, rather than a bacterial lymphadenitis or soft tissue infection, proper.

## 3. Discussion

*Sarcina ventriculi* is a gram-positive, anaerobic coccus with carbohydrate fermentative metabolism [9] and characteristic morphological features. Veterinary literature has reported *Sarcina* in the development of gastric dilation [10] and death of livestock. *Sarcina* has also been reported to be seen in feces of humans consuming a vegetarian diet [11]. Reports have shed light on associations with gastric dysfunction as evidenced by the presence of this microorganism in gastric biopsies of patients with delayed gastric emptying [12]. *Sarcina ventriculi* has also been described in patients with gastric perforation [6], emphysematous gastritis [7], and gastric adenocarcinoma [8]. Importantly, *Sarcina ventriculi* has also been reported in gastric specimens without pathological changes, suggesting that in some patients and in some clinical settings, these bacteria may represent commensal organisms rather than pathogens. It is challenging for pathologists to know the significance of *Sarcina* in clinical specimens, and as such, the presence of this microorganism should be reported to our clinical colleagues to allow for optimized patient management [13]. In some patients, bacterial proliferation and gastritis have been reported with signs of mucosal injury, concurrent diabetic gastroparesis, history of surgical scarring, or malignancies [14].

Our patient presented with epigastric pain and bloating. These presenting symptoms have been described in patients with *Sarcina*. In addition to these findings, vomiting and nausea are other reported presenting symptoms [8]. Upper endoscopy revealed signs of gastritis, duodenitis, and gastric outlet obstruction, fitting well with clinical features in other documented reports of symptomatic patients with *Sarcina*^8^. To our knowledge, one previous case of *Sarcina ventriculi* occurring in the setting of pancreatic adenocarcinoma has been reported by Ratuapli et al. [8]. This patient was status post pancreatico-duodenectomy. In our report, the extrinsic compression of the duodenum by the perigastric mass causing a partial gastric outlet obstruction and delayed gastric emptying provided favorable conditions, an acidic pH of the stomach and the presence of carbohydrates as nutrients, for the growth of *Sarcina*, with all of these factors correlating well to several published reports regarding the pathogenesis of this organism [3, 14, 15]. *Sarcina ventriculi* is typically histologically diagnosed from hematoxylin-eosin-stained sections taken from the mucosal tissue biopsies, but molecular methods such as polymerase chain reaction and sequencing of the 16S ribosomal RNA (rRNA) and the pyruvate decarboxylase gene, a unique metabolic pathway in S*arcina*, can also be used [12] to confirm the diagnosis.

The pathologic role of *Sarcina ventriculi* is debated as it has been identified in clinically asymptomatic patients as well as in patients with life-threatening conditions like emphysematous gastritis and gastric perforations. Treatment with antibiotics and proton pump inhibitors have been reportedly used in symptomatic individuals [8], while it may be reasonable to forego antibiotic treatment in asymptomatic patients [16]. Currently, there is no proven association of *Sarcina ventriculi* with pancreatic adenocarcinoma, but identification of these organisms warrants mention, as knowledge of their presence may provide a better global understanding of an individual patient's condition. *Sarcina ventriculi* organisms have been only rarely reported in cytology specimens, including urine cytology, esophageal brushings, and patients undergoing endoscopic ultrasound-guided biopsy evaluation for diagnosis and staging of gastric adenocarcinoma [17–19]. To our knowledge, this is the first reported case of *Sarcina ventriculi* in an FNA of metastatic pancreatic carcinoma in a perigastric lymph node. As mixed bacterial forms were present, and minimal accompanying inflammation was seen, carry-through organisms from the gastric lumen were favored.

## Data Availability

All data generated or analysed during this study are included in this published article.
